# Bottom-up perspective – The role of roots and rhizosphere in climate change adaptation and mitigation in agroecosystems

**DOI:** 10.1007/s11104-024-06626-6

**Published:** 2024-04-04

**Authors:** T. S. George, D. Bulgarelli, A. Carminati, Y. Chen, D. Jones, Y. Kuzyakov, A. Schnepf, M. Wissuwa, T. Roose

**Affiliations:** 1 The James Hutton Institute, Invergowrie, Dundee, DD2 5DA UK; 2 Plant Sciences, School of Life Sciences, University of Dundee, Invergowrie, Dundee, DD2 5DA UK; 3 Department of Environmental Systems Science, ETH Zurich, Universitätstrasse 16, 8092 Zurich, Switzerland; 4 The UWA Institute of Agriculture, & School of Agriculture and Environment, The University of Western Australia, Perth, 6009 Australia; 5 Environment Centre Wales, Bangor University, Bangor, Gwynedd LL57 2UW UK; 6 Department of Soil Science of Temperate Ecosystems, Department of Agricultural Soil Science, University of Göttingen, 37077 Göttingen, Germany; 7 Peoples Friendship University of Russia (RUDN University), 117198 Moscow, Russia; 8 Forschungszentrum Jülich GmbH, IBG‑3 (Agrosphere), Wilhelm Johnen Str, 52428 Jülich, Germany; 9 PhenoRob Cluster, Bonn University, Bonn, Germany; 10 School of Engineering, Faculty of Engineering and Physical Sciences, University of Southampton, Southampton, UK

**Keywords:** Plant roots, Rhizosphere functions, Climate change mitigation, Adaptation, Food security

## Abstract

**Background and Aims:**

Climate change is happening and causing severe impact on the sustainability of agroecosystems. We argue that many of the abiotic stresses associated with climate change will be most acutely perceived by the plant at the root-soil interface and are likely to be mitigated at this globally important interface. In this review we will focus on the direct impacts of climate change, temperature, drought and pCO_2_, on roots and rhizospheres.

**Methods and Results:**

We consider which belowground traits will be impacted and discuss the potential for monitoring and quantifying these traits for modelling and breeding programs. We discuss the specific impacts of combined stress and the role of the microbial communities populating the root-soil interface, collectively referred to as the rhizosphere microbiota, in interactions with roots under stress and discuss the plastic responses to stress as a way of adapting plants to climate change. We then go on to discuss the role that modelling has in understanding this complex problem and suggest the best belowground targets for adaptation and mitigation to climate change. We finish by considering where the main uncertainties lie, providing perspective on where research is needed.

**Conclusion:**

This review therefore focuses on the potential of roots and rhizosphere to adapt to climate change effects and to mitigate their negative impacts on plant growth, crop productivity, soil health and ecosystem services.

## Introduction

At the beginning of this century climate change was predicted to cause a rise in global average temperature of between 1 to 7 °C compared to pre-industrial levels by the end of the twenty-first century Such climate change is a consequence of unprecedented rates of greenhouse gas emissions into the atmosphere caused by global industrialization, notably raising the atmospheric pCO_2_ to levels (> 400 ppm) not recorded for over 800,000 years with impacts not only on temperature, but also global weather patterns and precipitation (IPCC 2023). Current projections are less optimistic at the lower boundary of this temperature change with predictions of temperature increases of between 2.6 and 4.8 °C over current levels by the end of century (The Royal Society 2020) and pCO_2_ could reach 800 ppm. According to current records, 2023 will be the 10th consecutive year when global temperature is on average more than 1 °C above pre-industrial levels and was globally the warmest year on record (prediction for 2023 is 1.2 °C above pre-industrial levels according to the UK Met Office, 2023). Climate change is clearly happening, and society has accepted that the temperature increase should only be restricted to 1.5 °C to limit negative effects, but unless strict mitigation measures are followed in the coming years, this aspiration seems extremely unlikely (IPCC 2023).

Even at the current level of global temperature increase, changing climate patterns affect agroecosystems (Muluneh 2021; Malhi et al. 2020). An increased frequency of extreme weather events (Pugnaire et al. 2019) will more often cause droughts, flooding, heatwaves, and reductions in winter frosts (Dempewolf et al. 2014; Calleja-Cabrera et al. 2020), affecting plants without adequate tolerance to drought, inundation, heat and those that require winter cold to complete their lifecycle (Simelton et al. 2013). In many parts of the tropics and subtopics it is predicted that more than three of these potentially severe impacts will concatenate leaving regions vulnerable to multiple and synergistic stress (UK Met Office, 2023). While the unpredictability of the environment will increase, it is predictable that these changes will have consequences on productivity, sustainability and conservation of biodiversity and other ecosystem functions. These changes will be most acutely felt at the current boundaries of the range of a given crop species, where some adaptations to this change will be possible through natural adaptation of populations (Exposito-Alonso et al. 2019; Jia et al. 2020). However, this will only be possible if sufficient adaptive phenotypic plasticity (Brooker et al. 2022) or genetic diversity exists in these boundary zones (Anderson and Song 2020), and will be contingent of whether breeding of new crop varieties will be quicker than the ongoing rate of change caused by climate change.

The effects of climate change on global crop yields will strongly depend on the geographic and climatic region and the potential beneficial impacts of raised pCO_2_ on increased carbon fixation and net primary productivity (NPP) by plants, also known as CO_2_ fertilisation. For example, Jägermeyr et al. (2021) estimated yields of wheat and maize to change between + 18 and -24% during the next few decades. For Northwest Europe, yields of C3 crops may increase as a result of more favorable temperatures and CO_2_ fertilisation, whereas yields in southern and eastern Europe are likely to severely decline due to drought and heat (Asseng et al. 2013). However, much uncertainty remains because such predictions of impact on yield rarely take into account the impact of climate change on pest and disease pressure and its interaction with abiotic stress. It is likely that crop yield and quality will be affected by changes in the prevalence, type and severity of pests and pathogens driven by interactions with the abiotic environment, particularly temperature and water availability (Newton et al. 2011).

Despite the many uncertainties caused by the multi-fold interactions of abiotic and biotic factors, the need to adapt crops to the changing climate is evident even now, and this will become a more pressing issue as we approach, and will exceed, the 1.5 °C limit and continue to increase pCO_2_ levels in the atmosphere. Initially much research has focused on aboveground adaptations of crops to increasing pCO_2_ levels (Zak et al. 2000; Kuzyakov et al. 2019), to extreme temperatures and droughts that affect water use efficiency (Peters et al. 2018; An 2022) or pollen viability and seed set, for example. We argue that many of abiotic stresses associated with climate change will be most acutely perceived by the plant at the root-soil interface and are likely to be mitigated at this globally important interface.

Plants have various root-based strategies to adapt and to compensate the negative effects of heatwaves, droughts, floods, salinity etc. Such adaptations may involve alterations in root architecture, anatomy, and physiology with effects on water and nutrient uptake efficiency (e.g. Hazman and Brown 2018 – drought imposed for 4 weeks without irrigation; Klein et al. 2020 – drought induced by withholding 50% irrigation; Li et al. 2022a; Deng et al. 2021). These adaptations likely involve interactions with physico-chemical and biotic factors in the rhizosphere (Hallett et al. 2022), the unique root-soil interface defined by and impacting on plant growth, development and health. Changes in soil temperature will affect interactions between plants and soil, most notably the microbiome (Ma et al. 2017; Ruan et al. 2023), while changes in water availability will be impacted at the root-soil interface by management practices such as fertilisation impacting the microbiome (Ruiz et al. 2020). It is, therefore, clear that impacts on the environment brought about by climate change will be played out belowground and that root and rhizosphere traits will be implicit in adaptation and mitigation to the change, potentially offering many of the solutions to the problem (Calleja-Cabrera et al. 2020).

In this review, we focus on the direct impacts of climate change on roots and rhizospheres, and root-soil interactions. The main climate change factors considered here are fluctuations in water availability (predicted by IPCC to be in the range of between 100% wetter in some global regions and 250% drier in others), particularly drought, and temperature increase (predicted by the Royal Society to increase by between 2.6 and 4.8 °C). In addition, we also consider the role of elevated pCO_2_ and greater C fixation by plants (predicted to increase from 400 ppm to as much as 800 ppm by century end). We consider which belowground traits will be impacted and discuss the potential for monitoring and quantifying these traits for modelling and breeding programs. We discuss the specific impacts of combined stresses as well as the role of the rhizosphere microbiota and plastic responses to stress as a way of adapting plants to climate change. We then go on to discuss the potential that modelling has in understanding this complex problem and suggest the best belowground targets for adaptation and mitigation to climate change. We finish by considering where the main uncertainties lie, providing perspective on where research is needed. This review therefore focuses on the potential of roots and rhizosphere to adapt to the climate change effects and to mitigate their negative impacts on plant growth, crop productivity, soil health and ecosystem services.

## Below-ground components relevant for climate change adaptation and mitigation

**Fig. 1:**
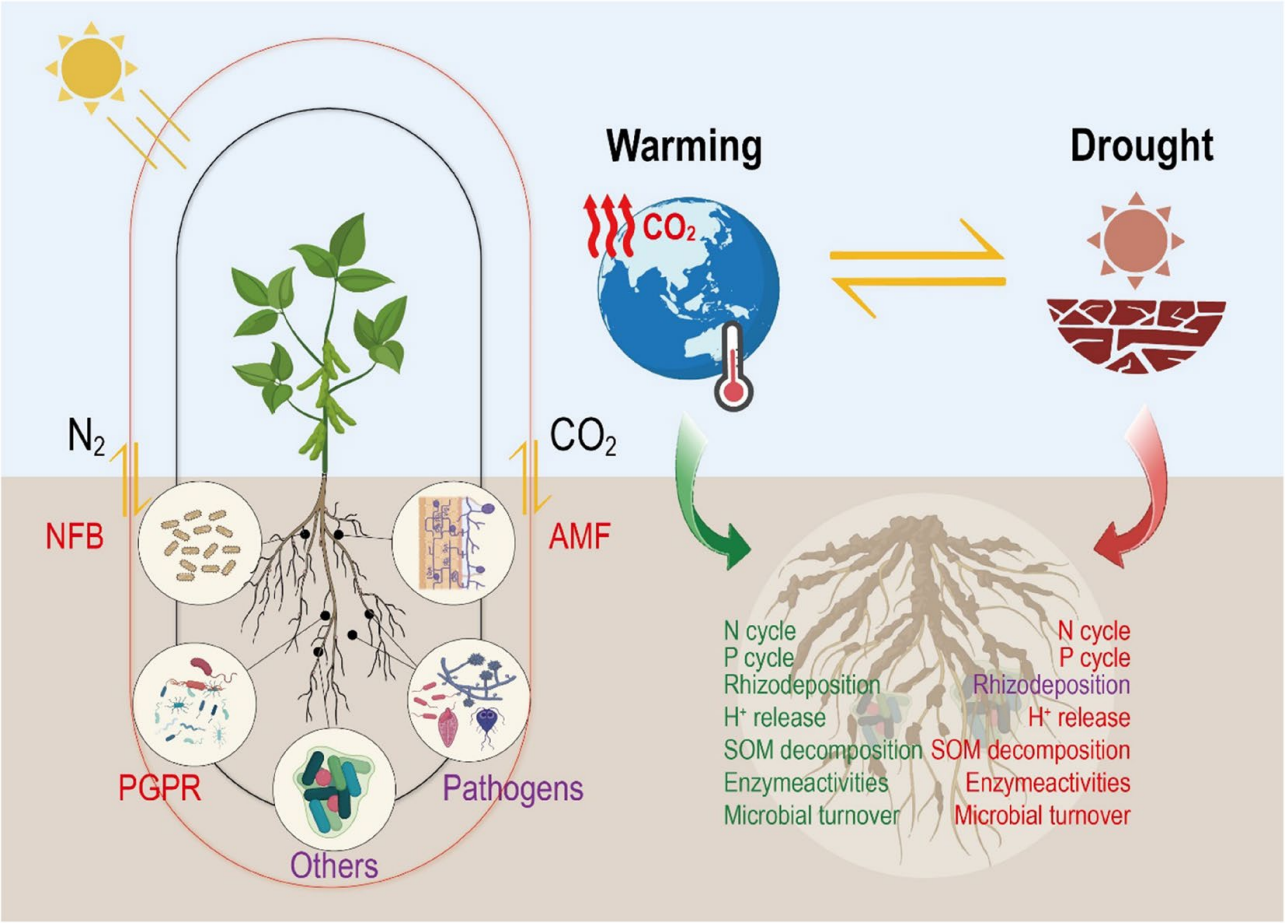
Impact of drought, warming and elevated pCO_2_ by climate change on rhizosphere microorganisms and functions. The overall impact of climate change significantly affects rhizosphere microorganisms with generally negative effects on N_2_-fixing bacteria (NFB), arbuscular mycorrhiza fungi (AMF), and plant growth promoting rhizobacteria (PGPR) (in red text) and both positive and negative effects reported for pathogens and other microorganisms (in purple colour). Plant-microorganism interactions mostly positively respond to warming (text in green), while they mostly respond negatively to drought stress (texts in red, or in purple for rhizodeposition when both positive and negative effects were reported)

Plant roots and the rhizosphere around roots are the main below-ground components relevant for climate change adaptation and mitigation. York et al. (2016) distinguished between the abiotic rhizosphere, characterized by changes in soil structure and depletion or accumulation zones of water and solutes, and the biotic rhizosphere characterized by rhizodeposition and microbial communities (see Fig. 1). While in reality it is difficult to separate the abiotic and biotic factors and both are of course tightly linked and hence more integrative.Studies to specify these linkages are needed if we were to be able to predict the system function in the changing climate using modelling.

Root architecture and anatomy are key determinants of plant resource acquisition as they determine the volume of soil that is accessible to the plant as well as the ease by which resources can be accessed. When roots grow into soil, they change soil structure mechanically or via rhizodeposition, thereby changing the soil’s hydraulic and chemical properties and inducing water potential and solute depletion or accumulation profiles. Plants have developed mechanisms to maintain good contact with the soil despite decreasing water potentials. These mechanisms are clearly manifested in the formation of a stable layer of soil particles adhering to the root surface—the so called **rhizosheath** (George et al. 2014; Holz et al. 2018). Soil particles adhere to the root surface being enmeshed with root hairs and mycorrhizal hyphae and glued to it by mucilage, glucoproteins and extracellular polymeric substances (EPS) (Agnihotri et al. 2022). The entanglements of soil particles with rhizodeposits, bacterial EPS, hairs and mucilage is likely to attenuate shrinkage (compared to the case of a root in a nutrient solution). **Mucilage**, released from the root tip, plays a crucial role maintaining the continuity of the liquid phase during drying (Carminati et al. 2013). Due to its high viscosity, low surface tension and water adsorption capacity, it maintains the hydraulic root connection to the soil matrix (Benard et al. 2019).

Plant roots also affect the rhizosphere microbiome where microbial activity is always much greater (for activities of some enzymes increased by 10 times) than in the bulk soil (Fig. 1) and this zone is accepted as one of the most important hotspots of microbial activity on the planet (Kuzyakov and Blagodatskaya 2015). In addition to this increased activity there is selection and recruitment of specific microbial species and often a reduction in diversity *per se* (Bulgarelli et al. 2015; Escudero-Martinez et al. 2022), but an increase in functional diversity and a unique set of trophic interactions involving bacteria, fungi, archaea, protists and nematodes (Mezeli et al. 2020). While, such changes in diversity, function and interactions will be dealt with later, initially we will focus on the changes in microbial activity in the rhizosphere. Microbial activity can be assessed by the dynamics of pools and by their functions. Regarding the pools (proportion of active microorganisms) and depending on the approach, the portion of active microorganisms in the rhizosphere range from 3–5% (Kuzyakov and Blagodatskaya 2015), whereas it is not greater than 1% in the root free soil. Because of greater C and energy availability, bacteria in the rhizosphere and bulk soil have opposite strategies for dormancy (Ling et al. 2022). The dormancy of bacteria in bulk soil is mainly based on spore formation (sporulation) (Ling et al. 2022), which is an energy saving strategy suitable especially for long dormancy periods. In contrast, the dormancy of rhizosphere bacteria is mainly based on the toxin-antitoxin system, which is more energy demanding but allows very fast reactions to the input of new or limiting resources.

Compared to the microorganisms in the bulk soil, rhizosphere microorganisms are likely to remain active for longer under climate change scenarios leading to greater impacts on function (Fig. 1) because i) compared to rapid exhaustion of available organics by warming in bulk soil, rhizosphere microorganisms will likely be getting excess C resources deposited by roots under elevated pCO_2_, and ii) the rhizosphere remains wetter for longer under drought, because of increased wettability through released mucilage, leading to a larger water content.

Microbial death is an overlooked dynamic, because nearly all microbial ecology studies focus on living microbial biomass and composition, growth and functions. There have been studies showing that the addition of fertilisers can cause microbial death with the level of death depending on the level of soil saturation (Ruiz et al. 2020). These mechanisms depend on the climate effects such as warming, drought, and elevated pCO_2._ Microbial death can occur by autolysis, long-term starvation, osmotic burst, chemical or physical damage, freezing, heating, irradiation, as well as killing by other organisms: by predation by nematodes and protists and lytic phage infection (Sokol et al. 2022; Camenzind et al. 2023). The myriad changes caused by climate change (elevated pCO_2_, temperature, water availability etc.) and their effects on the complex food web found in soils are likely to be varied and difficult to predict, therefore adding another layer of complexity into understanding the impact of climate change on the activity and function of the microbiome in the rhizosphere. This is worthy of a review in its own right, but is beyond the scope of this particular manuscript and so is only alluded to here as being important.

Summarizing, the two main climate change effects: warming and drought – strongly affect microbial activity, composition and life-death cycles. Rhizosphere microorganisms are more susceptible  to warming and drought *per se*, however the more predictable rhizosphere environment regarding C and energy availability, as well as water and nutrients provides a better location for microbes to overcome stresses compared to bulk soil. It is important to also consider the overriding impacts of abiotic stress caused by climate change on alterations of the complex food web and trophic interactions in which the microbiome sits and the effects of stress on life and death cycles of the plants themselves, with particular emphasis on the dynamics of root dieback and regrowth as a response to stress. Thus, the interplay between crop and soil management can have both, positive and negative, impact on soil microbiome and predicting the outcome is a complex problem that would benefit from interrogation using next generation modelling.

## Impact of climate change on root and rhizosphere traits and functions

### Water availability/drought

One aspect of climate change already noticeable in agriculture over the past years is the increasing unpredictability of rainfall patterns that increases the likelihood of drought and flooding events (Bevacqua et al. 2022). Of particular concern are more frequent drought events that would negatively affect crop productivity, even in humid climates from temperate Northern Europe to tropical Africa. Drought manifests itself in various forms and severities, from short intermittent to severe prolonged drought, and its effect furthermore depends on whether the drought period coincides with the more sensitive crop establishment or reproductive stages and on the temperature during the drought. Drought is most often related to heat stress in agricultural systems, not only because of reduced ability to regulate both water use and leaf temperature, as will be discussed later, but also due to increased soil temperature which can affect many aspects of root growth and function and is mitigated somewhat with depth.

Root and rhizosphere traits will be of key importance to mitigate effects of more frequent water shortages and shall therefore be explored first before also discussing potential impacts of rising temperatures. It is well established that one of the key responses of roots to water stress is to promote growth of roots at depth to capture water deeper in the profile and in some cases redistribute water to other parts of the root system by hydraulic lift (Lynch 2013). This deep rooting phenotype is also a potentially effective way of roots avoiding the impacts of heat stress caused by increased soil temperatures. Beyond this there will be impacts of drought on root anatomical traits, on which we will focus here. Moreover, it is likely that in some circumstances drought stress will be so extreme that root growth will cease, and dieback of roots will occur as plants remobilise resources to maintain photosynthesis and survival. Because of this the importance of precision soil management to avoid the extreme drought conditions is likely to grow exponentially as the soil amendment timing in dry climates is predicted to have a large impact on crop productivity (McKay Fletcher et al. 2022; Fletcher et al. 2021).

#### Impacts on roots and root hairs

Severe soil drying causes root shrinkage (Khare et al. 2022). Duddek et al. (2022) showed that root hairs shrink under relatively wet soil conditions (at water potentials less negative than -0.1 Mpa), which is followed by the shrinkage of root cortex (at water potentials around -1 MPa). Earlier work (Carminati et al. 2013) suggested that roots shrank after transpiration was reduced, indicating that shrinkage is a consequence of limitation in water availability. However, the work by Duddek et al. (2022) showed an early root hair shrinkage, which might imply that the rhizosphere, and in particular root hairs, might be sensors of water limitations, impacting both water and nutrient transport from drying soils into the roots. Water stress also has significant impact on root anatomical structure, such as alteration of the number and size of the metaxylem vessels (Prince et al. 2017 – imposed 5–40% volumetric water content [VWC]). Plants can decrease the number of new metaxylem cells and increase pith cells to enhance water uptake capacity under water-limited conditions (Mangena 2018).

The growth of root hairs shows opposite patterns to the main roots. Root hairs grow longer and denser in soils with large porosities and thus on roots with smaller contact to the soil matrix. This is not surprising, as root hairs become increasingly important when water flow and nutrient transport toward the root surface becomes limited, i.e., when the liquid contact between the root surface and the soil decreases. The importance of root hairs for nutrient uptake (particularly for solutes with limited mobility) has been well documented. On the contrary the role of root hairs for water uptake remains controversial. For barley, hairs provide an advantage in dry soil 0.1 cm^3^ cm^−3^ VWC) conditions both in the field and controlled conditions (Marin et al. 2021), but the effects were absent in maize (Cai et al. 202110^1^–10^3^ kPa matric potential). One explanation is that maize has shorter and less dense hairs than barley (Burak et al. 2021). Another explanation is that in maize root hairs shrink at relatively high water potentials (around -100 kPa, Duddek et al. 2022) and thus might lose their capacity to extract water (and maybe nutrients) in relatively wet soils. Root hair shrinkage depends on soil water potential and hair age. It occurs when hairs lose turgidity—i.e. at the turgor loss point. When the soil water potential reaches the turgor loss point, shrinkage starts. As hairs age, their turgor loss point is likely to become less negative leading to earlier shrinkage. The variability of the turgor loss point of hairs across species and soil conditions is not known.

Similar to root hairs, roots shrink as the soil water potential decreases and they lose contact with the soil (Carminati et al. 2013). Cortical shrinkage occurs after hair shrinkage, with hair shrinkage being the first of a series of root responses to soil drying. Root shrinkage leads to a reduced capacity to extract water from the soil. However, shrinkage might occur only after water and nutrient availability, as well as stomatal conductance, is severely reduced, and may therefore be a consequence rather than the cause of water limitation. Clearly, with increased temporal variation in soil moisture content with climate change, the ability of plant roots to be resilient to such shrinkage and the ability to have an elastic or plastic response to water availability will be critical.

#### Impacts on water dynamics in the rhizosphere

Water use regulation depends on the interactions between soil drying and plant hydraulics. At a critical soil moisture, water supply from the soil can no longer sustain the transpiration demand of plants (Sinclair 2005). The threshold soil water content depends on soil hydraulic properties such as the unsaturated hydraulic conductivity and the water retention curve, as well as on plant properties (Carminati and Javaux 2020). Among these the root surface area active in water uptake, which is related to root system structure is important, as are above-ground factors that drive water demand, such as canopy conductance and atmospheric conditions (temperature and humidity). Root and leaf traits will impact the thresholds of soil water limitation, as reviewed in Cai et al. (2022). Key traits include root length and root hydraulic conductance and how this changes with decreasing water potential through processes such as cavitation. Additionally, plants can modify the properties of the rhizosphere (porosity, soil structure, wettability) in different ways discussed below, all of which may be useful for adaptation to climate variability.

Root mucilage also has the benefit of altering soil physical conditions to increase the ease of extraction of water (Naveed et al. 2017a, b) and to stabilize soil structure (Agnihotri et al. 2022). Increasing the root hydraulic conductivity will also be crucial to aid water uptake and this could be achieved by enhancing membrane transporter (aquaporin) responses and membrane fluidity (Calleja-Cabrera et al. 2020). Reducing xylem vessel diameter might trigger an earlier stomatal closure (Richards and Passioura 1989), but may attenuate the risk of xylem embolism and enhance the expression of aquaporins in root membranes (Hacke et al. 2010).

A different aspect of plant adaptation to drought is the speed with which a plant may recover from drought after water supply is restored. After drying and subsequent rewetting, mucilage delays the rewetting of the rhizosphere and temporarily limits the recovery of root water uptake (Kroener et al. 2016). Reactivation of root water uptake after drought is related to multiple factors. Beside mucilage wetting, plant tissues need to rehydrate. It is likely that cells do rehydrate after rewetting, as shown for root swelling after rewetting (Carminati et al. 2013). However, extremely dry conditions can cause embolisms in the root xylem vessels, which are then not easily refilled with water. Recovery from embolism is controversial and reactivation of the hydraulic function of these root segments is unlikely. Therefore, new root growth becomes essential for restoring water uptake.

#### Impacts on rhizosphere microbiome

Plants release a plethora of organic compounds to the rhizosphere in a process called rhizodeposition. Rhizodeposition directly impacts the chemical, physical and biological properties of the rhizosphere. Rhizodeposition components, consisting of root exudates, secretions, mucilage, dying root hairs and sloughed off cells, and root released enzymes, respond differently and specifically to water limitation. Due to greater osmotic pressure caused by drought (30% water content relative to field capacity), the passive loss of organic compounds from root cells, called exudates, will increase (Sanaullah et al. 2012). To simplify soil penetration and to produce moisture films around the root, as well as to increase the contact to the mineral soil particles, roots increase the release of mucilage under drought (Holz et al. 2018—6% volumetric soil water content). Root hair death, as well as shrinkage (Duddek et al. 2022), will be strongly accelerated under limited moisture. Summarizing, the flux of most components of rhizodeposition from roots into the soil will increase under water limitation (but not under complete water absence) (Deng et al. 2021). Considering the decreased nutrient uptake by plants under drought, the C and energy costs per unit of utilized nutrient will strongly increase, potentially limiting CO_2_ fertilisation and increased yield. However, some of the rhizodeposits may have disproportionate effects on nutrient availability and balance some of this decline.

Soil microbes, such as rhizobacteria, have the ability to secrete exo-polysaccharides, alter endogenous phytohormones and antioxidants, and a diverse cocktail of compounds e.g., sugars, amino acids, and polyamines, volatile organic constituents, dehydrins, and heat shock proteins (Kaushal and Wani 2016). Through altering physiological and biochemical processes in plants, the microbes could help plants to mitigate drought stress by preserving plant growth, membrane stability, and enzyme constancy and effectively controlling water and mineral uptake by increasing the root surface area (Kumar and Verma 2018; Vacheron et al. 2013). For example, a Plant Growth Promoting Bacteria (PGPB) strain, *Pseudomonas putida* GAP-P45, decreased reactive oxygen species (ROS) accumulation and reduced the activities of all antioxidant enzymes in *Arabidopsis thaliana* seedlings (Ghosh et al. 2018), and thus improved the plant resistance to water-stress imposed by the addition of polyethylene glycol (PEG).

This greater microbial activity in the rhizosphere is confirmed by increased microbial respiration (measured as CO_2_ efflux) under drought (Deng et al. 2021). Another parameter of microbial activity – activity of extracellular enzymes – increase over the short term (< 1 year) but will be nearly completely recovered over longer periods (Canarini et al. 2021). Consequently, microbial community shifts under drought imposed in the field (10% VWC imposed by rainout shelter) include taxa having higher activity and enzyme production under water limitation.

Members of the phylum Actinobacteria recently gained a prominent role in dissecting the impact drought as occurrence of this stress triggers an enrichment of the proportion of these bacteria compared to other phylogenetic groups populating the microbiota at the root-soil interface (Xu and Coleman-Derr 2019). Interestingly, this drought-triggered enrichment appears to be conserved across plant lineages as evidenced by results gathered with 18 different species belonging to the class Monocotyledonae (Naylor et al. 2017). Actinobacteria belong to the group of so-called monoderm (or Gram positive) bacteria, which appear to be better adapted to arid soil conditions compared to diderms (or Gram negative) bacteria (Naylor et al. 2017). From a genetic standpoint, Actinobacteria define a group of the plant microbiota with a relatively low functional diversity within families, as revealed by high-throughput comparative genomics (Bai et al. 2015) Yet, functional conservation and metabolic adaptation to abiotic condition alone cannot explain a microbial enrichment being predominant in the root and rhizosphere microbiota while less marked in unplanted soils (Xu et al. 2018; Santos-Medellín et al. 2017 – imposed severe drought until senesce and leaf curling was apparent. 5% VWC). A prediction of these observations is that Actinobacteria enrichment under drought stress may confer an adaptive advantage to their host plants under drought. Consistently, experiments conducted under laboratory conditions indicated that inoculation of representative strains of Actinobacteria conferred growth promotion to Sorghum seedlings only following exposure to drought treatment (Xu et al. 2018 – removal of irrigation at the 9th week of growth). From a molecular standpoint, this growth promotion capacity appears to be conferred by several mechanisms, including production of phytohormones, osmolytes and osmoregulatory substances (Ebrahimi-Zarandi et al. 2023).

**Fig. 2:**
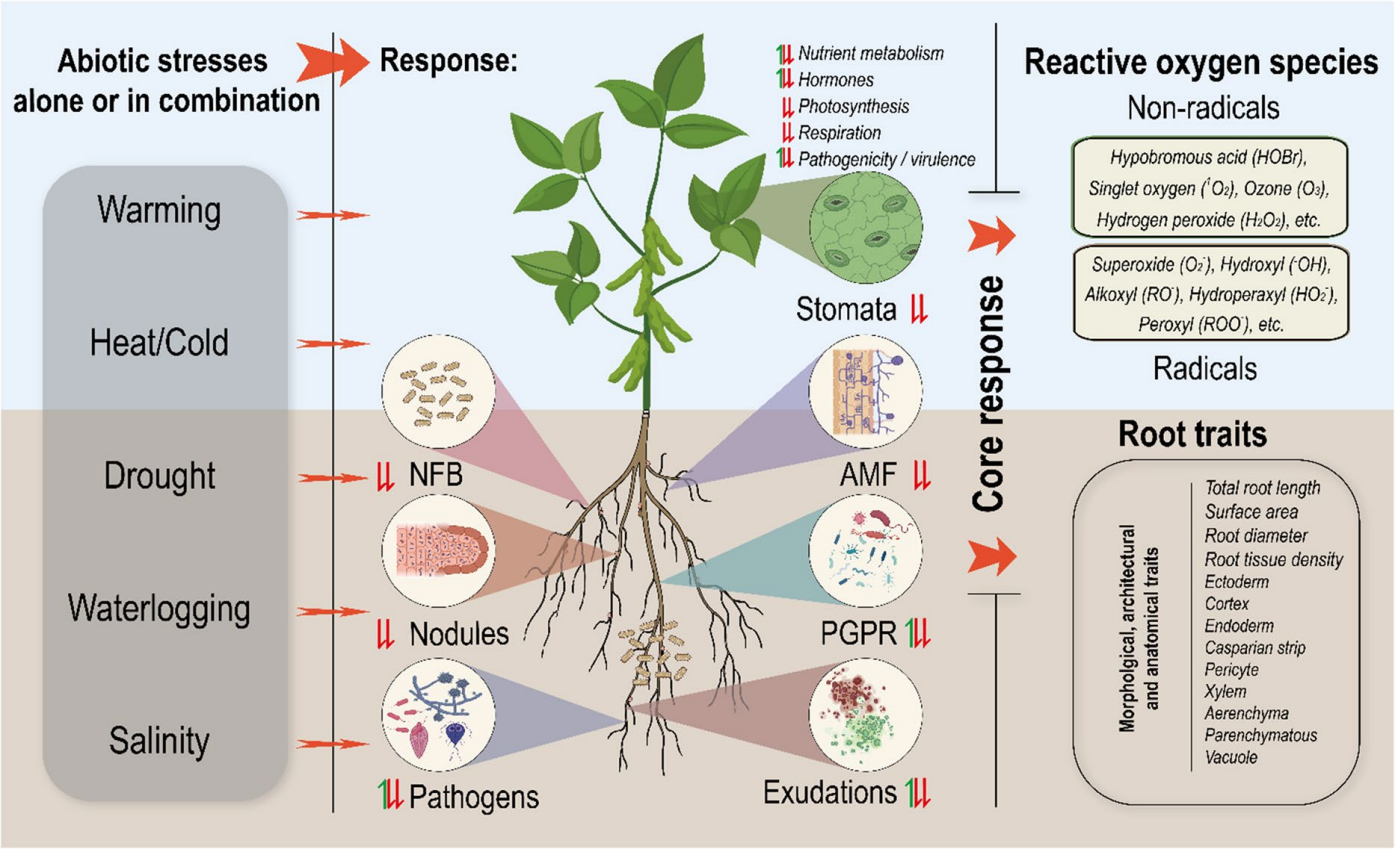
Impacts of abiotic stress alone or in combination on the function of roots and rhizospheres. Combined stresses often have greater effect on the plants than the sum effect of individual stresses. Abiotic stresses directly or indirectly affect plant stomatal closure in leaves, root structure, nutrient uptake, rhizosphere microbes and secretions from roots and microbes. The core responses of plants to abiotic stress include the regulation of reactive oxygen species (ROS) in shoot and root. Different abiotic stress may induce different response in root morphological traits and anatomical traits. NFB, N_2_-fixing bacteria; AMF, arbuscular mycorrhiza fungi; PGPR, plant growth promoting rhizobacteria

Combined stresses often have greater effect on the plants than the sum effect of individual stresses. Abiotic stresses directly or indirectly affect plant stomatal closure in leaves, root structure, nutrient uptake, rhizosphere microbes and secretions from roots and microbes. The core responses of plants to abiotic stress include the regulation of ROS in shoot and root. Different abiotic stress may induce different response in root morphological traits, anatomical traits, and interactions with the microbiome including arbuscular mycorrhiza fungi, rhizobium and plant growth promoting rhizobacteria (Fig. 2).

### Impacts of heat on belowground processes

Soil temperatures are likely to increase in line with atmospheric temperatures and this will be most acutely felt in the uppermost few centimetres of the topsoil, whereas temperature increases will be attenuated somewhat with depth. Increases in maximum temperatures as a result of climate change could take some below ground processes beyond critical thresholds, but they also pose an additional problem, especially if they coincide with periods of drought, with the need to keep stomata open to cool leaves at odds with the need to conserve water. In theory increasing temperatures should make many of the belowground processes more efficient or faster, with the rates of biological, biochemical and chemical processes being increased. For instance, root growth rate, nutrient turnover and movement and enzyme activities will be increased and are unlikely to reach their maximum efficiency within current temperature bounds. Where we are most likely to see impacts of temperature increases will be on the rhizosphere microbiome.

The rhizosphere microbiota has an ability to manage its metabolism to overcome changing temperature and preserve their membrane and enzyme stability by establishing a cascade of heat and cold shock proteins. High-temperature stress causes protein denaturation, which is mitigated against by trehalose through formation of a gel-like web to save plants from dehydration (Shameer and Prasad 2018). Cold-adapted microbes found in high-altitude agro-ecosystems, have a large potential to assist plants in alleviating unfavorable climatic conditions. A wide group of phylogenetically unrelated bacteria, encompassing the genera *Alcaligenes* sp, *Arthrobacter* sp, *Bacilus* sp, *Delftia* sp, *Methylobacterium* sp, and *Pseudomonads* sp isolated from heat-tolerant plants improved wheat growth and development under heat stress (Yadav et al. 2015). To avoid enzyme denaturation by warming, rhizosphere microorganisms produce isoenzymes, having the same functions, but with higher temperature stability at the costs of lower substrate efficiency (= higher Km) (Razavi et al. 2017).

Rhizosphere microorganisms also provide beneficial conditions to overcome short-term heat waves and to adapt to long-term warming compared to microbes in bulk soil. These beneficial conditions are connected with the water content of the rhizosphere being more tightly regulated, aided by mucilage release (Benard et al. 2019), and much greater C and energy availability in the rhizosphere compared to bulk soil (Gunina and Kuzyakov 2022), especially considering that rhizodeposition increases with soil warming (Wei et al. 2019).

Most rhizosphere bacteria accelerate their growth rates due to warming and elevated pCO_2_, especially members of the phyla Bacteroidetes, Gemmatimonadetes (Jin et al. 2022 – 800 ppm eCO_2_). Consequently, their abundance increases, and more rhizodeposits will be consumed and mineralized to CO_2_. Conversely, members of the Actinobacteria and Acidobacteria decrease their growth rates, and consequently, their abundance in the rhizosphere (Ruan et al. 2023 – imposed 500 ppm eCO_2_ with + 2 °C). Despite that bacterial taxa are phylogenetically conserved, climate change modifies the strategies of over 90% of species, partly confounding the initial phylogenetic pattern (Ruan et al. 2023).

Changes in microbial death rates in the rhizosphere caused by warming is a complex issue. The metabolic rates of microbes increase by warming and thus raise the demand for resources and energy. This increased demand leads to a decrease in the microbial population, as resources become limited and microbial populations become stressed. Higher temperatures also increase susceptibility to environmental stresses, such as desiccation, UV radiation (at the soil surface), oxidative stress by reactive oxygen species (ROS) (Yu and Kuzyakov 2021), which further reduce microbial populations in the rhizosphere. Microbial death can be caused by the production of toxic metabolites or the release of toxins from other organisms as well as predation by higher trophic groups such as nematodes and protists and impacts of phages. Bacteria killing by phages, however, decrease as phages are less adapted to warming (Williamson et al. 2017), but the impacts of changes in pCO_2_, temperature and drought on higher trophic organisms, such as bacterial feeding nematodes, is variable with metanalysis showing that there is limited impact of rising temperature on abundance and activity, but more consistent positive impacts of drought and pCO_2_ on bacterial feeding nematodes (Zhou et al. 2022). Clearly, subtle changes in soil temperature could have important impacts on the structure and function of the rhizosphere microbiome and therefore the response of associated plants to climate change.

## Combinations of stress and plasticity in traits to fluctuations in stress

### Plasticity to fluctuations of stress

**Fig. 3:**
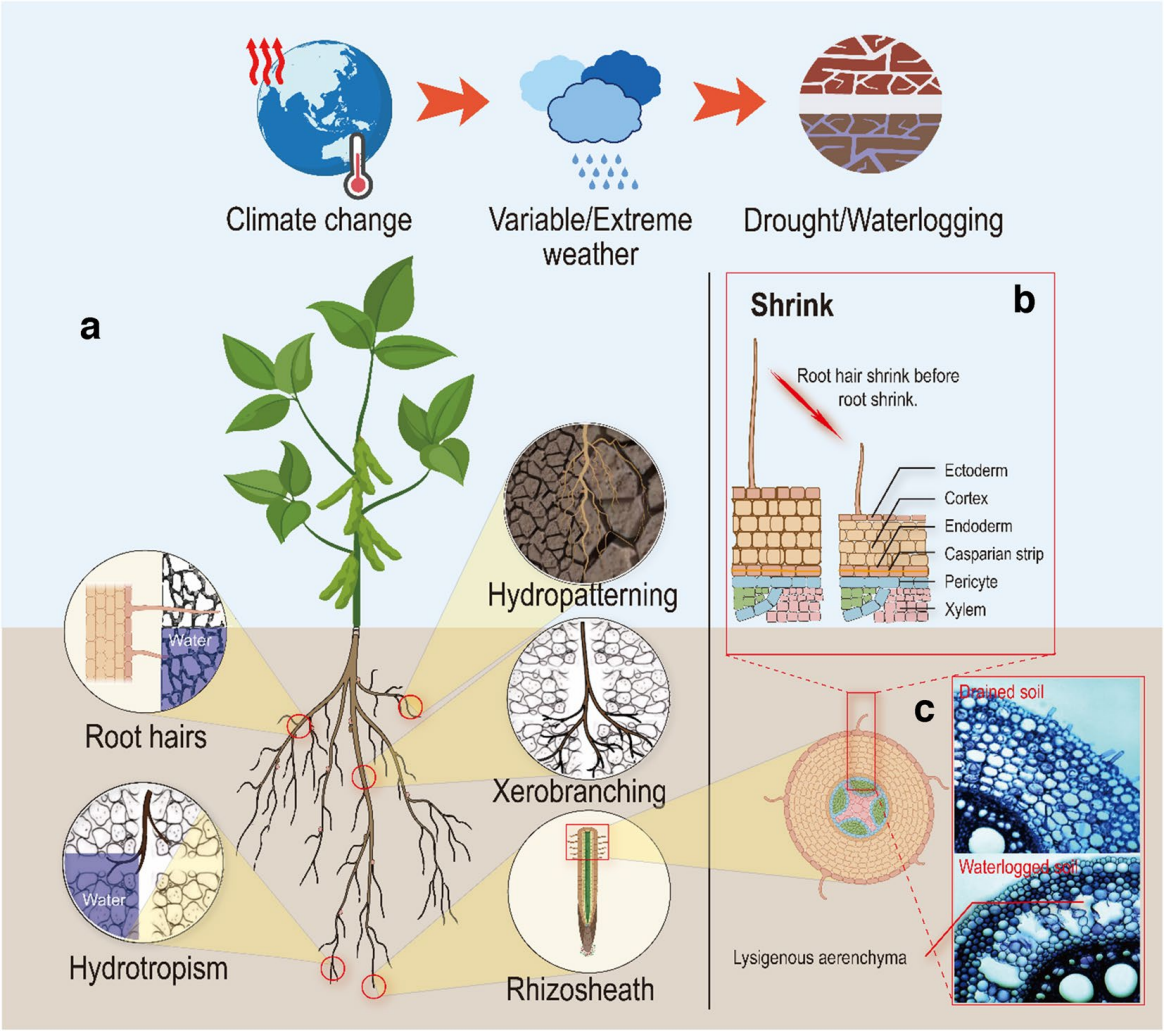
Root trait plasticity to stress fluctuations under climate change. **a**. Climate change causes drought and waterlogging to plants due to variable and/or extreme weathers. In order to adapt to drought and waterlogging, plants often alter root system architecture, hydrotropism, hydropatterning, xerobranching, and root/root hairs/rhizosheath shrink. **b**. Water deficit causes root hair shrinkage, followed by root cortical cell dehydration, resulting in root shrinkage. **c**. Waterlogging causes the root parenchyma to breathe anaerobically, producing large amounts of ethylene and reactive oxygen species, as well as programmed cell death. Lysigenic aerenchyma formed during lignification to store oxygen to combat waterlogging (maize root section as an example, Drew et al. 2000)

Climate change likely means more irregular precipitation, so frequent periods of water deficit or drought would alternate with occasional heavy rains and flooding (Fig. 3). Therefore, there will be more extreme drying-wetting cycles in the rhizosphere and one may ask how well roots are adapted to such changes. For instance, how quickly can roots adapt to soil drying and recover when water becomes available? This concept can be termed adaptive plasticity which is defined as the ability of a genotype to change its phenotype in response to variation in environmental conditions in a way that maintains the organism’s function and sustains plant productivity and yield. Such plasticity has been studied in rice under alternating drought and flooding cycles and genotypic differences in the proliferation of lateral roots during dry cycles and rapid aerenchyma formation during subsequent flooding were demonstrated (Suralta et al. 2018). Furthermore, these differences could be associated with quantitative trait loci (QTL) for aerenchyma formation (Niones et al. 2013 – waterlogging to 20% VWC) and lateral root plasticity (Niones et al. 2015 – waterlogging to 20% VWC), demonstrating that root plasticity could potentially be selected for in crop breeding programs. A genuine understanding of adaptive plasticity is essential to test this assumption, and to assist with current efforts – including targeted crop breeding – to develop crop cultivars capable of adapting to the increased amplitude of fluctuations in climatic conditions expected from climate change (Brooker et al. 2022).

Plants have developed several mechanisms to adjust their growth according to the spatial variability in soil water availability and could be useful targets for adaptive plasticity (Fig. 3). Soil drying and the consequent increase in soil penetration resistance, possibly increase the pressure on the root tip and enhance the secretion of mucilage. This would result in a phenotypic plasticity in this trait. Hydrotropism is the preferential growth of the root tip toward wetter regions. Hydropatterning is the preferential branching of lateral roots towards the soil matrix of roots growing in soil macropores with asymetric contact with soils. Xerobranching is the lack of root branching in root segments that have no contact with the soil matrix. These mechanisms show the root growth plasticity and have been mainly investigated in artificial soils in well controlled lab conditions with the scope of identifying the underlying molecular mechanisms. There are only few investigations of such mechanisms on root growth in the field. de Moraes et al. (2020) found for soybean grown in field site with a Brazilian Oxisol that drought resulted in a combined effect of mechanical and hydric stresses that reduced elongation rate, resulting in reduced rooting depth as well as root length density. An important question is the implication of root growth plasticity for time variable water conditions as well as whether they are reversible or irreversible (Sjulgård et al. 2021). For instance, as the soil dries, water availability and possibly also the asymmetry of water availability around (some) roots is likely to change, impacting the degree of hydrotropism and pattern mechanisms. If xerobranching is triggered by water availability rather than by a loss of contact, it would result in reduced branching in dry soil layers, and possibly into a more cost-effective root growth in wetter soil regions.

A different but nevertheless very important aspect of crop productivity in water-limited environments is the speed of recovery following a drought. It has been shown that rapid compensatory growth following a drought can avoid yield losses (Hoogenboom et al. 1987). Whether this recovery is associated with reversal of transcriptional or biophysical responses or simply by the growth of new roots is open to conjecture. Moreover, recent studies have demonstrated the role of stress memory and therefore epigenetics in the response of plants and their root phenotypes to repeated or cyclical stress (reviewed by Jacques et al. 2021 for drought stress). This work demonstrates the transcriptional and physiological response of plants to repeated stress is different to that of a single stress event of equivalent magnitude; it also demonstrates the importance of the rhizosphere microbiome in coping with this. This highlights the need to consider the complexity and cycles of stress in real environments in future research (Liu et al. 2022) and how repetition of multiple stress factors and combinations thereof play out in the true response of roots to climate change related stress.

### Plant responses to combined stresses

Abiotic stresses often occur in combination leading to stronger effects that are often at least additive, if not antagonistic, and this further diminishes yield and quality parameters. Few studies have investigated combinations of abiotic stresses, especially for non-model species.

Plant responses to several abiotic stressors are distinct and cannot be inferred directly from responses to individual stress conditions (Mittler 2006, Bouain et al. 2019 and Vescio et al. 2020). Key pressures are noted by Mittler (2006), the majority of which are abiotic, to interact in various ways, and are all impacted by climate change. The transcriptome, proteome, and phenotypic levels of these plant responses to stress can all be observed to alter with combined stress (Mittler 2006; Ahuja et al. 2012; Ghosh and Xu. 2014).

**Fig. 4:**
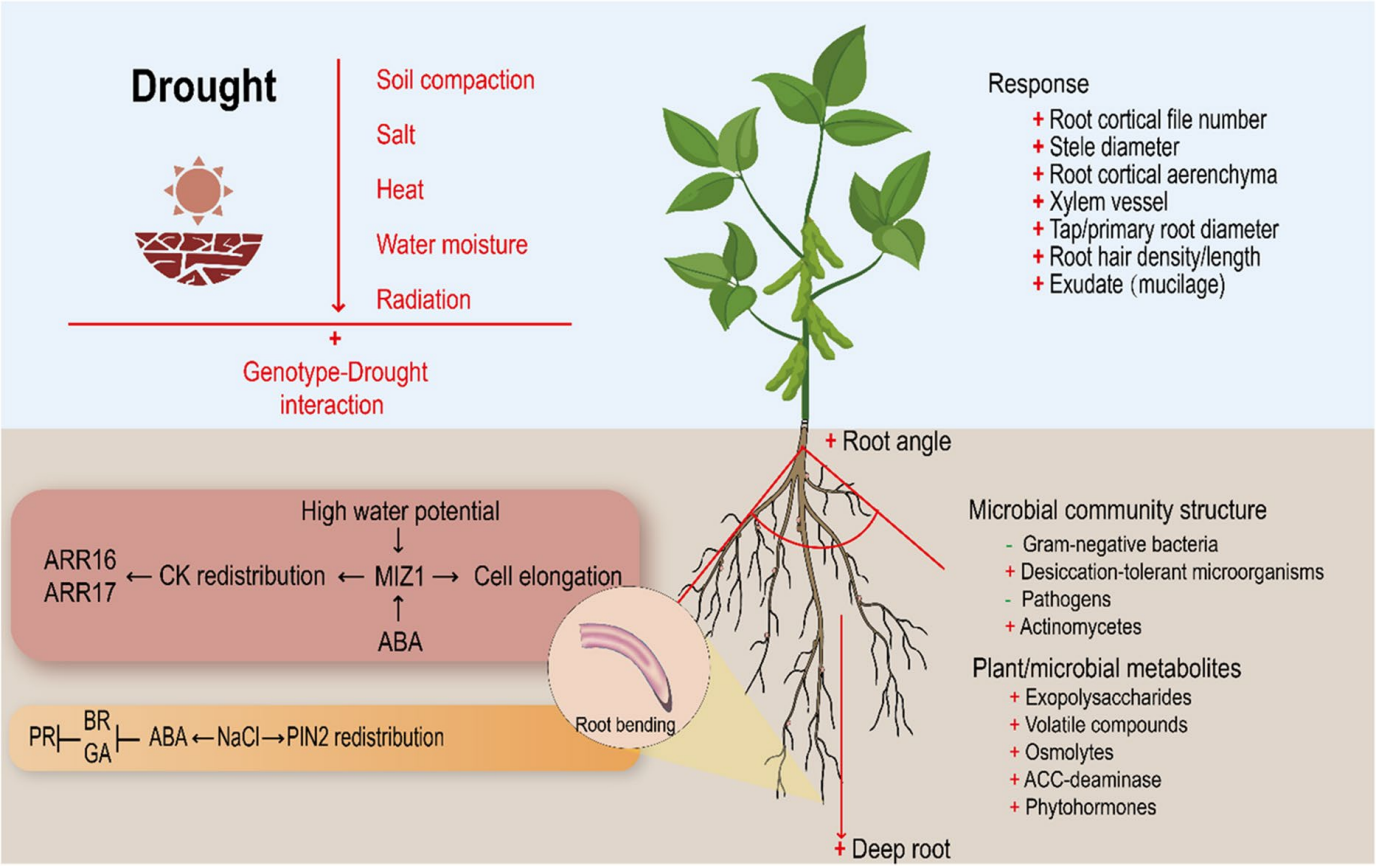
Root and rhizosphere response to combined stress associated with drought and heat. Drought could lead to soil compaction, salinization, high temperature and local water moisture and radiation. These stresses and their interactions determine root plasticity. ABA, abscisic acid; BR, brassinosteroids; CK, cytokinin; GA, gibberellin; PR, plant root; TFs: PIN2, MIZ1, ARR16 and ARR17 (Correa et al. 2019; Karlova et al. 2021)

There may be a universal plant response that helps combat abiotic stress in general. Importantly, some of these core plant responses will have knock-on effects on the structure and functionality of roots, rhizosphere, and microbial interactions. Production of antioxidative compounds to combat the impact of ROS appears to be a core response included in these plant responses. Despite this universal reaction, when several stresses are present, many of the reactions to each will act in opposition to one another. For instance, drought-induced stomatal closure to conserve water will diminish a plants ability to adjust leaf temperature under high ambient temperatures (Rizhsky et al. 2002 – imposed 23 °C to 44 °C at 65–70% relative water content), as well as reduce nutrient uptake through mass flow. Because energy and resources are required for plant adaptation to abiotic stress through changes in root system architecture and functions, nutrient deprivation could pose a serious problem to plants attempting to cope with heat, drought or salinity stress and this could be exacerbated by nutrient dilution caused by elevated atmospheric pCO_2_ and increased carbon fixation by plants (Fig. 4).

Combinations of stresses have impacts on root system architecture, adventitious root formation and morphological root features, such as aerenchyma formation (Shabbir et al. 2022), which are not predictable from an additive response to the individual stress. This has been demonstrated when considering combinations of multiple nutrient stresses (Bouain et al. 2019) and with combinations of a range of abiotic stresses associated with climate change, such as drought, heat, salinity and waterlogging (Shabbir et al. 2022). A number of studies demonstrate that this antagonism leads to the response of the plant, or in the rhizosphere, akin to that of a response to the dominant stress as seen with nutrient limitation, but also seen with combined heat and drought stress (Vescio et al. 2021). For instance, experiments conducted with maize mutants impaired in phytosiderophore production and iron uptake revealed that this mutation provokes an enrichment of Actinobacteria in the rhizosphere comparable to the one observed when plants are exposed to drought stress (Xu et al. 2021). This suggests an interdependency between nutritional (i.e., iron) and drought stresses leading to a common microbial enrichment in the rhizosphere. Interestingly, experiments conducted under laboratory conditions revealed that exogenous application of iron to sorghum seedlings exposed to drought stress (removal of irrigation at 9th week of growth) failed to trigger both a rhizosphere enrichment or the growth promotion of individual Actinobacterial strains (Xu et al. 2021), possibly indicating that ‘iron stress cues’ override ‘drought stress cues’ during microbiota assembly. It is also demonstrated that root types are affected differentially by individual and combined stress, with seminal roots specifically responding to combined stress (Vescio et al. 2020). Vescio et al. (2020) show that maize seminal root growth was inhibited by combined heat (32 °C) and drought (30% available water content), when unaffected by the individual stresses in isolation. With simultaneous beneficial changes to primary lateral roots, such as increased length and reduced root diameter, they suggested this represented a shift away from areas of poor water availability to those with replete water at depth.

 Combined stress has an impact on the compounds exuded from roots and is different from the impact of the individual stresses in isolation. For example, Tiziani et al. (2022) showed that combined stress of drought (30% available water capacity) and heat (32 °C) on exudates from maize roots was unique, in comparison to impact of the individual stresses in isolation. This change in root exudates is likely to cause changes in the rhizosphere microbiome as was shown for maize where specific compounds upregulated in exudates were responsible for selection of specific microorganisms (Vescio et al. 2021; Tizani et al. 2022) and for tomato exposed to combined salt and Verticillium stress (Flemer et al. 2022). Moreover, exudate compounds with putative plant growth promoting properties were evidenced in rhizosphere microorganisms facilitated by stress changes (Vescio et al. 2021). It is also clear that more intimate interactions with mycorrhizal fungi, for example, will have impacts on regulating the plants response to abiotic stress in isolation or in combination (Begum et al. 2019). Begum et al. (2019) suggest that the fundamental alteration of the plant phytohormone profile, mineral nutrient uptake and upregulation of the plant antioxidant system, provide the plant with an innate resistance to multiple stress. Interestingly, for some plant growth promoting endophytes their abilities were only realised under combined abiotic and biotic stress (Flemer et al. 2022). Therefore, it can be reasonably assumed that the unique plant physiological and molecular response to combined stress will lead to changes in traits below ground and foster specific interactions with rhizosphere microorganisms which will have impact on rhizosphere functions. Interestingly, this response pathway appears to be a two-way path, with inoculation of specific microorganisms in the rhizosphere causing changes to the combined stress (heat and drought, 40 °C, drought induced by PEG) response of the plant (Bilal et al. 2020; Begum et al. 2019).

The adaptation of plants to a combination of different abiotic stresses will, therefore, require an appropriate response customized to each of the individual stress conditions involved, as well as tailored to the need to compensate or adjust for some of the antagonistic aspects of the stress combination and will be dependent on the developmental stage when the stress is perceived, the frequency and variation in stress and the range of trophic interactions impacted by the stress response.

## Potential root and rhizosphere ideotypes for climate resilient plants

Root ideotypes for specific environments have been described for specific targets: the steep, cheap and deep root system for efficient N and water uptake (Lynch 2013), the topsoil foraging ideotype for efficient P acquisition, and an intermediate ideotype for K (White et al. 2013). Shelden and Munns (2023) proposed a salt-tolerant root ideotype that would include halotropism to avoid highly saline soil patches as well as root anatomical changes to restrict sodium uptake. However, climate change will result in more variable and *a priori* unknown weather patterns as well as the occurrence of combined multiple stresses.

Adapting crops to drought and heat stress is certainly not a novel concept developed in response to anticipated climate changes. Crop production in the semi-arid tropics and Mediterranean climates has always been exposed to drought and research has addressed the issue over the past 50 years or more. What is new, as a result of climate change, is that the frequency and intensity of drought is spreading further into temperate climates against a backdrop of elevated pCO_2_, forcing agriculture to adapt. In the breeding context this would mean drought traits will have to be considered for the first time in the formerly favorable temperate climates, whereas Mediterranean and dry-continental climates may need to consider the full arsenal of drought-related traits that may have previously been reserved for crops in arid and semi-arid regions. Selection of a root system for higher temperature tolerance could be possible. Root system plasticity itself is discussed as a trait. Suralta et al. (2018) provided evidence that components of root plasticity in rice are genetically controlled and therefore conceivable targets in crop breeding, however, further research is needed to expand to other crops (Schneider and Lynch 2020), see also Section “Combinations of stress and plasticity in traits to fluctuations in stress”. Alternatively, management methods such as the use of mixtures with complementary root systems are currently investigated (e.g. Demie et al. 2022). Traits have been proposed in the past, but complexities of translating root populations for root traits, has limited progress to date. However, the urgency of challenges ahead necessitates finding practical solutions and the following sections explore to what extent recent advances in phenotyping methodology can provide solutions and where a better conceptual understanding of traits will be needed before practical solutions can be provided.

In the context of climate change in temperate climates, where some of the world’s largest crop yields are currently achieved, it is expected that short intermittent droughts, such as those seen in Europe in 2022, will occur more frequently (IPCC 2023). Maintaining these high crop yields can only be assured if carbon assimilation is facilitated by maintaining optimal stomatal density and opening during periods of water deficit. This would best be achieved through the continued supply of water from roots to leaves and traits which allow rapid exploration of deeper and, therefore, more moist soil. Traits of interest in this regard are a faster root descent rate (Kulkarni et al. 2017), an increased proportion of deeper roots (Lopez et al. 2019) and the ability to plastically increase root growth rates in deeper soil in response to drying topsoil (Hoogenboom et al. 1987), prolific root branching to explore a larger soil volume (Khatun et al. 2021), and improved penetration into hard and dry soils, possibly as a consequence of thicker root axes with a large proportion of stele (Klein et al. 2020).

As drought increases in severity and/or duration, plants need to re-balance their water demand in accordance with decreasing water availability. Adjusting the growth and development between above- and belowground plant parts through limiting shoot growth and diverting resources to enhance root growth is one way to better balance water supply and demand. The resulting increase in the root:shoot ratio is one typical response observed in drought-stressed plants (Yamaguchi and Sharp 2010; Xu et al. 2013 – drought imposed with PEG). Conserving water can furthermore be achieved by stomatal closure at the onset of stress, which has been shown to be helpful to save water for later critical stages (e.g. flowering or grain filling) and is thus associated with greater yield (Vadez 2014). In addition, plants need to protect the photosynthetic machinery of the chloroplasts from toxic elements and reactive oxygen species (ROS) by producing heat shock proteins, osmoprotectants to delay the onset of senescence for as long a period as possible. On the other hand, under water limiting conditions, partial stomatal closure reduces water consumption, attenuates the drop in leaf water potential, and eventually contributes to sustained water losses. Understanding and manipulating the Abscisic Acid (ABA) signaling pathways to optimise stomatal opening for the given environmental conditions should be a target. While traits involved in stomatal regulation are found in the shoot or in signaling between the shoot and the root, it will also be important to consider water uptake capacity of the root system and understand the interactions at the root-soil interface. On one hand, increasing the capacity of roots to extract water has the added advantage of maximising nutrient acquisition, which is critical for the effective metabolism of carbon fixed by photosynthesis and reduce accumulation of sugars and sink strength feedback issues. The initial response to drought of increasing soil exploration by developing deeper and highly branched roots will provide more water and nutrients. On the other hand, maximising crop water uptake will lead to earlier soil drying and also increase the exposure of the crop to potentially toxic elements in saline conditions. Following this reasoning, Vadez (2014) proposed that the role of roots in conferring drought tolerance is not only to increase water extractability from the soil profile, but also to regulate water use and flow, trigger stomatal closure and potentially save water. This concept establishes an important link between root hydraulics and stomatal closure. Relevant root anatomical architectural traits impacting root hydraulics and the link to water use and stomatal regulation are discussed below together with a brief background on theory of water flow in soil and plants.

As already highlighted, maintaining canopy temperatures within acceptable ranges will require continuing water uptake to sustain transpirational cooling and a root system exploring deeper soil is likely the most important adaptations, as discussed above. Furthermore, temperatures in the topsoil may increase to levels far beyond optimal for growth of species and genotypes (Calleja-Cabrera et al. 2020). Again, the most promising strategy for plants to avoid excessively high temperatures in the root zone is to proliferate roots at depth (Füllner et al. 2012—20 °C to 10 °C surface to base of profile) where an optimized root distribution across the soil profile will assure access to water and nutrients during droughts and short heat waves (Kautz et al. 2013). Maintaining access to water during drought and heatwaves will also allow crop genotypes, particularly those C3 species whose carbon fixation capacity is not saturated under current conditions, to maximise the advantage of elevated pCO_2_ to growth and NPP.

Beyond the roots there is an extended phenotype that can also be considered part of the ideotype, and includes the rhizosphere microbiome. For instance, Plant Growth-Promoting Rhizobacteria (PGPRs) and arbuscular mycorrhizal fungi (AMF) can provide their host plants with enhanced access to mineral nutrients, adapt to abiotic stress conditions and mitigate climate stress (Sebai and Abdallah 2022; Lugtenberg and Kamilova 2009). These communities are not randomly assembled from the surrounding environments, rather are the result of a multi-step selection process controlled at least in part by the plant itself (Bulgarelli et al. 2013; Edwards et al. 2015). This allows the microbiome to be selected for in a crop ideotype and should be considered along with the root and shoot traits highlighted above.

One of the biggest limitations to progress in this area is the lack of accurate and efficient root and rhizosphere phenotyping tools limiting understanding of the root and rhizosphere response to climate change, particularly to heat stress. Notwithstanding this, recent progress in the development of root related methodologies has significantly enhanced our capacity to measure, visualize and model roots and rhizospheres (Chen et al. 2015; Li et al. 2022b; Oburger and Schmidt 2016). Traditional soil coring, shovelomics and trench profiling can be used as complementary techniques to minirhizotrons under field conditions (Bilyera et al. 2022). In addition to the destructive measurements through excavating root systems and rhizosphere, non-destructive techniques (X-ray CT, magnetic resonance imaging (MRI), neutron radiography (NR), Ground penetrating radar (GPR), zymography) have been developed for high-throughput visualization and quantification of root-soil interactions (Oburger and Schmidt 2016). However, it is important to acknowledge that most root and rhizosphere related phenotyping tools do not give a full description of the root system and its interaction with the soil and are laborious with limiting throughput, making them difficult to apply to the size of populations required to perform genetic studies and selection. Given the inherent complexity of root phenotypes, generating an ideotype is exceptionally challenging, and requires interdisciplinary efforts, ranging from mathematics to root biology, to genetics and agronomy, in multiple environments at both laboratory and the field scales (Li et al. 2022c).

## The potential of modelling in developing root and rhizospheres for climate resilient plants

The complex interactions between root systems and their soil environment, and the difficulties associated with visualizing and measuring these interactions, make studying the plant–soil continuum a challenge. Current development of structure–function root models offers excellent opportunities to characterize rhizosphere interactions and determine factors governing root–soil interactions, particularly impacts of temperature, water availability and elevated pCO_2_. Root models can be used to simulate root growth and rhizosphere biological, physical and chemical processes in a spatially and temporally varying environments (De Dorlodot et al. 2007) and are invaluable tools in scenario testing where we would like to consider multiple stress, environment and management. Thus, by integrating rhizosphere and growth data, simulation and modelling studies are capable of linking predictive laboratory techniques with field studies, allowing researchers to strategically predict, evaluate and target beneficial root traits or ideotypes for specific growth environments in particular under climate change scenarios. Moreover, modelling has the power to address simultaneous variation in multiple traits in multiple environments, which cannot be achieved in biophysical experiments and field trials. A general modeling framework has been proposed as a starting point for developing new rhizosphere models to address current gaps by linking and coupling soil ecology, physics, and chemistry, and considering rhizosphere microorganisms (Kuppe et al. 2022a).

How modelling has contributed to the study of rhizosphere processes has been outlined in several recent review papers on the more technical aspects of modelling and will not be repeated here. One of the most recent review papers offering a broad review of different modelling methodologies and discussing which techniques are useful in which experimental context is by Ruiz et al. (2021). The authors distinguished three contemporary techniques in plant-soil interactions modelling, using a distribution based, architecture based, and image-based representation of roots. The research question determines the choice of root representation. For example, in situations likely to occur under drought stress, such as limited and heterogeneous water, architecture-based models allow to mechanistically represent phenomena such as root water uptake compensation and hydraulic redistribution. Image-based models have a high computational demand, but for some problems they are the preferred options, e.g. when small-scale interactions between roots are of importance. Upscaling from single root to root system and field scales for real-world problems is discussed in the review of Roose et al. (2008), while Kuppe et al. (2022b) provide an extensive review of single root models. Three dimensional hydraulic architectures of crops and how to link these to more computationally efficient and hence also more widely-used 1D or 2D models are described by Vanderborght et al. (2021). Modelling also supports crop phenotyping (Tracy and Wright 2020) as prior to field trials modelling allows for *in silico* experiments on a lot larger scale than possible in real life, a promising route towards climate-smart root phenotypes. Integrating knowledge across different systems and disciplines, e.g., the approach of Vanderborght et al. (2021) provides a link between model approaches (a) and (b) as described in Fig. 1 of Ruiz et al. (2021).

**Fig. 5:**
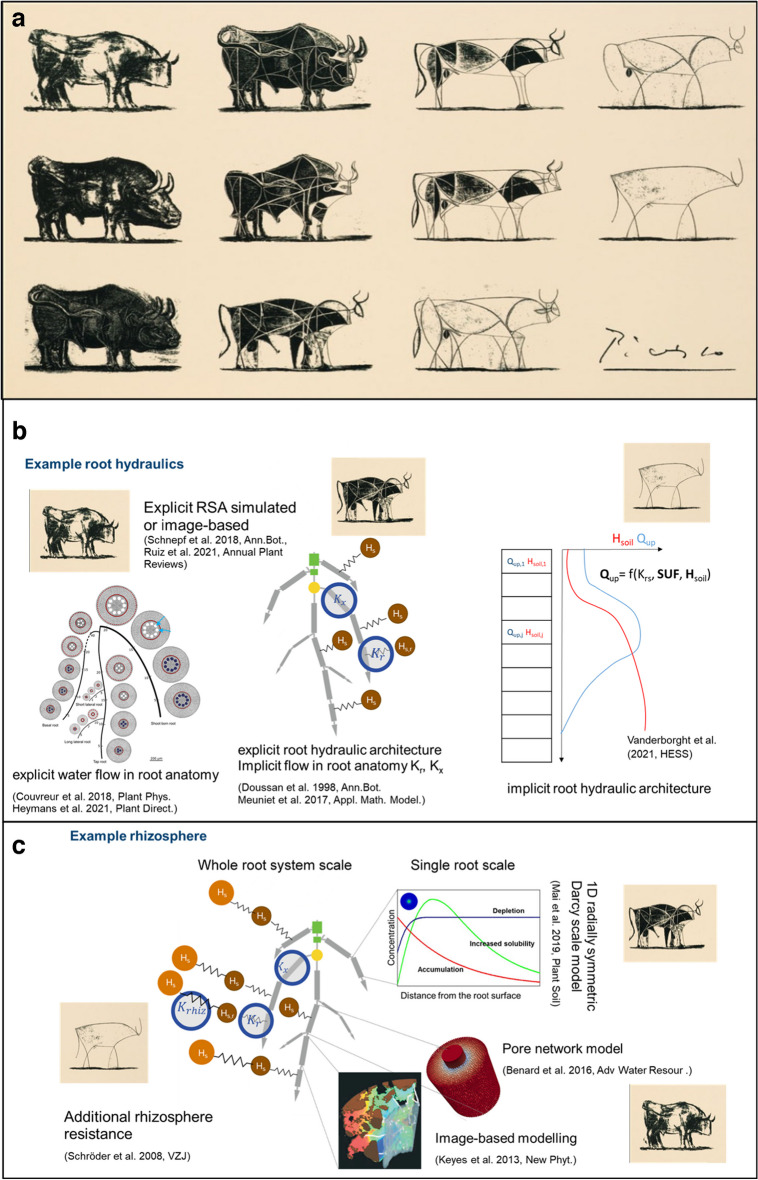
**a**. Picasso bulls (reproduced with permission from www.artyfactory.com) . **b**. Example: three levels of complexity of modelling root water uptake. The highest level of complexity explicitly considers the root architecture as well as the root anatomy, the medium level of complexity replaces the complex root anatomy with two scalars, radial conductances (K_r_) and axial conductances (K_x_). The lowest level of complexity implicitly considers root architecture for every soil layer by a function for root water uptake that depends on two properties that can be derived from the root system architecture (RSA), the equivalent root system conductance (*Krs*) and standard uptake fractions (SUF). This step is explained in detail in Vanderborght et al. (2021). **c**. Three levels of complexity of a rhizosphere model. The highest level of complexity models the soil around the root using image-based models or pore network models. At medium complexity, we can use 1D radially symmetric Darcy scale models where the soil is treated as continuum, but we still solve one rhizosphere model for each root segment of the root system. At the lowest level of complexity, this can be replaced by adding an additional rhizosphere resistance for water flow towards the root surface, which is a function of the soil and root water potentials, this function can be derived from the single root models. This step is described in detail in Vanderborght et al. (2023)

It will be important to identify only the most important physical and biological processes for effective root and rhizosphere models. The challenge is that some of these processes can take place at very different scales and thus it is difficult to distinguish which processes are important and when. This often results in thinking that including “everything” in the model is productive whereas this can only create an illusion of completeness, i.e., including everything in one model makes the model more complex and data hungry. The challenge really is to know when enough detail is included parsimoniously. Philosophically speaking, the modeler is faced with the similar question that Picasso posed for his drawing “when does bull become a bull?”; see Fig. 5.

Just like for Picasso, the modeler’s approach depends on the observer (for some problems the very first abstract bull might be enough, but for others not) and the purpose of the model within the wider scientific discovery process. Moving across spatial scales with modelling has its own technical challenges, i.e., it is not entirely obvious how to translate results/modelling that is important for example on the soil pore to the field or even regional scale as not all processes and features on the pore scale are important on the field scale. This is when sophisticated mathematical techniques, such as homogenisation (Pavliotis and Stuart 2008), become useful as they allow for formal and rigorous upscaling of modelling across spatial and temporal scales. The method of homogenisation has its origins in multiple timescale analysis (Hinch 2008) which was then generalised to 3D spatiotemporal problems such as fluid flow through periodic porous media and today has been extended to general reactive flow and transport problems in (non-periodic) porous media.

Recently, the mathematical challenge has been how to represent severe weather events that are more likely to increase with the changing climate in a computationally efficient manner. For example, it is intuitively obvious that 4 mm rain falling over 10 min period would have a different impact on soil and rhizosphere than 4 mm rain falling over 24 h period and yet most of the soil/rhizosphere models assume that the rainfall events can be averaged over 24 h + periods. On one hand time averaging like this is necessary as it is not computationally efficient to run simulations with 1 min resolution in anticipation of the large rainfall event, but equally, the impact behavior of such extreme events needs to be adequately and accurately captured as they can have major effect on fertilizer and crop behavior in the soil. Performing greater time and space resolution combinatorial computer simulations is still in its infancy (McKay Fletcher et al. 2022) as the access to high performance computing facilities can be a hurdle. Thus, it is often the combination of complex models with simpler (1D or 2D) models that is most efficient as this interaction allows for proofing and validation of the simpler models thereby building confidence in all mathematical model building steps that can appear to be opaque to non-specialist modelers.

Modeling and interacting with data require cautious navigation. If a model has enough unconstrained input parameters, it should fit any given data well. For instance, a straight line can always be drawn through two data points with 100% accuracy. However, it is surprising to observe the frequent use of complex models with 10 + parameters to explain only 3 to 5 data points. This tendency is likely influenced by incomplete data, emphasizing existing knowledge gaps (Amelung et al. 2020). We would like to stress that it is important to keep the modelling honest and explain uncertainties and data inconsistencies rather than pretending that the model is “perfect” as no model ever is. Currently, data availability for soil/rhizosphere modelling is ever increasing, from 3D and chemical imaging techniques, to geophysical methods to visualise processes in opaque soil to drone and satellite observations. This will take the field from a data-poor situation to a data-rich situation, creating new opportunities such as hybrid mechanistic and data-driven modelling, as well as new challenges, requiring larger multidisciplinary teams to integrate all streams of their work with modelling behind a common goal. The more processes are considered, the more important is a balanced view of the different processes and their interactions. When looking at small-scale rhizosphere processes, root growth is often neglected although it can be quite important for the development of the rhizosphere (Schnepf et al. 2022). Similar to weather forecasts, models can now be used to predict agriculturally interesting variables, being continuously informed by current data. This approach can, for example, be used to predict the optimal timing for nitrogen fertilizer application (Fletcher et al. 2021, 2022), an example to help climate change mitigation.

Overall, several recent reviews highlight modelling approaches for plant-soil-rhizosphere interactions. Until now, they have not been used systematically to address climate change adaptation and mitigation. Now is the opportunity to start using them for this purpose. The challenge is to do it with full experimental integration within the above-described scientific discovery path.

## Future priorities to help harness roots and rhizospheres for climate change mitigation and adaptation

### Understanding signaling and the interactome.

Plants produce a myriad of carbon compounds and many are released from their roots, influencing the composition and function of the rhizosphere microbiome in a very dynamic way (Liu et al. 2021). Carbon additions to soil alter the environment and affect microbial recruitment, growth and function (Xu et al. 2020) and in cohort with root traits such as root hairs (Koebernick et al 2017; 2019), or mycorrhizal hyphae (Zhang et al. 2018; Jiang et al. 2021). Climate change is likely to affect the quality and quantity of these rhizodeposits. Elevated pCO_2_ and temperature will have large impacts on the amount C fixed by plants and therefore what is available for rhizodeposition, while associated stress may impact the quality of the compounds produced. Indirectly, these rhizodeposits can alter the chemical and physical environment of the rhizosphere in which the microbes grow and reproduce (Naveed et al. 2017a, b, 2018, 2019). They also act as an energy source for these mostly heterotrophic bacteria and fungi. However, components of rhizodeposits also act as specific signals which influence specific functions of microbes, such as fructose activating bacterial phosphatase enzyme production and altering organic P mineralisation (Zhang et al. 2020) and nitrification inhibiting compounds directly effecting the expression of ammonia oxidizing genes and reducing conversion of ammonium to nitrate (Subbarao et al. 2012). More recently it has been shown that a range of plant hormones are also lost from the root into the rhizosphere and have impacts on microbial assemblage and function (Lu et al. 2021). Similarly understanding the impact on microRNA is still in its infancy. Moreover, the microbiome can feedback to the plant through rhizosphere signals altering its physiological and genetic response. This makes for an exceptionally complicated environment that varies from the bulk soil in many different ways. These rhizosphere interactions can be considered as a rhizosphere interactome, which has a key role in regulating plant stress tolerance. Approaching our understanding of plant microbe interactions from a novel perspective by treating it as a holobiont regulated by its interactome will likely provide important breakthroughs in understanding at this boundary of disciplines. Moreover, understanding the impact of climate change on these interactions is urgent and critical to deliver resilient and adapted crop genotypes. Development of novel and cutting-edge techniques in rhizosphere imaging of root rhizodeposits, high-throughput platforms for phenotyping root rhizodeposit signatures in large populations and unique and powerful genetic populations to interrogate these traits, gives us a novel and unique opportunity to push forward the boundaries of understanding. Add to this, established expertise in rhizosphere modelling and pipelines in rhizosphere microbiome metagenomics, rhizosphere stable-isotope probing and expertise in root-soil physical interactions the community is well poised to take the field beyond the state-of-the-art. The integration of all these fields of research has the potential to allow us to decipher the regulation of rhizosphere signals and feedback loops, which are yet unknown.

It is becoming evident that plants actively shape the microbial community inhabiting the root-soil interface (Bulgarelli et al. 2015; Robertson-Albertyn et al. 2017) and this is likely manifest in the rhizosphere interactome. Our understanding of these complex interactions will become clearer through first detecting and quantifying the plant and microbial exudates as well as their effects on gene transcription and translation. This will be greatly enhanced by advances in analytical chemistry and analytical immunology, such as capillary electrophoresis MS (CE-MS) and monoclonal antibody arrays, leading to an ability to measure a more complete metabolome that can be related to the genome and transcriptome of the plants and the metagenome of the soil. This can be combined with novel techniques such as metatranscriptomics which can provide insight into the specific expression and translation of genetic loci in the rhizosphere. Moreover, cutting-edge techniques such as different imaging mass spectrometery approaches will further allow direct visualisation and quantification on specific chemical exchanges in the rhizosphere interactome. Evidence from mammalian digestive and marine ecosystems demonstrates that polysaccharide (PS) complexity drives cognate microbial diversity via evolution of highly specialized carbohydrate active enzymes (CAZymes) for glycan degradation. These effects are less well understood in the rhizosphere but recent research suggests that analogous processes are important. The study of the rhizosphere interactome is in its infancy and focus in this area will generate novel understanding of this critical zone in global ecosystems and foster significant development in the area.

### Designing the root-soil interface

The root-soil interface defines a distinct microhabitat for a community of microorganisms whose taxonomic composition and function is markedly distinct from unplanted soil (Terrazas et al. 2016) and is the arena for the rhizosphere interactome to play out. These plant-microbial assemblages define a wide range of interactions encompassing both parasitism and mutualism (Escudero-Martinez and Bulgarelli 2019) at different levels of intimacy such as symbiotic relationships, including rhizobia and mycorrhizae, endophytic microbes thriving within the root corpus and free living organisms. The deterministic nature of interorganismal relationships at the root-soil interface is particularly attractive for translational applications, as it sets the stage for the development of optimum plant-microbe combinations for given soils (Schlaeppi and Bulgarelli 2015). Despite the fact that microbiota applications to increase plant performance have successfully been benchmarked under experimental conditions, in-field applications still suffer from poor predictability (Li et al. 2022c) and even less is known under the influence of climate change including elevated pCO_2_, temperature and an altered stress environment. This likely reflects the lack of a comprehensive understanding of the recruitment cues of the microbiota and their modulating factors, including climatic modifications.

As crop wild relatives of modern varieties have evolved under marginal soil conditions, their microbiota gained centre-stage in basic and applied science as an untapped resource for crops’ adaptation to the environment (Pérez-Jaramillo et al. 2016; Raaijmakers and Kiers 2022). Interestingly, a footprint of this differential evolutionary pressure is represented by the differential enrichment of members of the phyla Actinobacteria and Bacteroidetes in the microbiota of domesticated and wild genotypes, respectively in multiple plant species (Pérez-Jaramillo et al. 2018). This taxonomic diversification, combined with advancements in crop genetics, enables the identification of host genetic determinants of the microbiota at the root-soil interface as recently exemplified in monocots (Escudero-Martinez et al. 2022) and dicots (Oyserman et al. 2022) alike. In turn, these discoveries may expedite the development of novel varieties benefitting from microbiota associations. Chief towards the achievement of this task will be a precise understanding of the impact of abiotic stress caused by climate change on determinants of the microbiota and how this integrates with the complex food web in which it resides, in addition to the overriding impacts of climate change on the plants growth, phenology and stress related rhizodeopisiton.

### Future approach to breeding crops with better root systems

In order to better understand the mechanisms and evolutionary effects underlying ‘crop species' adaptation to environmental stress, large-scale genomic, phenomic, and ecological data must be combined (Li et al. 2019; Shim et al. 2021; Al-Hajaj et al. 2022). Likewise, such approaches can focus on mitigation of climate change with emphasis on carbon sequestration to soils and optimising the nitrogen cycle in the rhizosphere. In both cases, this should be done with a below-ground focus. In order to narrow down the search for stress tolerance genes and support environmental ideotype breeding, it is possible to use geographic and agro-ecological data, future climate predictive modelling, trait-based ensemble modelling, crop simulation, and trait-based modelling (Redden 2013, Rötter et al. 2015; Paleari et al. 2022; Yan et al. 2022). Additionally, the use of landscape genomics, the integration of genetic information with geographic data on sampling positions, has proved successful in discovering selection signatures (Allendorf et al. 2010; Schoville et al. 2012). New combinations of belowground trait genes for adaptation to climate associated stress will increase the diversity of the cultivated crop.

Plant breeding can be sped up using well-established methods such as marker-assisted and genomic selection, high-throughput phenotyping, and speed breeding (Ahmar et al. 2020; Pandey et al. 2022; Camerlengo et al. 2022). The development and selection of crops with improved traits, including rhizosphere microbiome traits for future target conditions with increased variable and extremes will be driven by genomic tools in the next phase of plant breeding (Redden 2013; Escudero-Martinez and Bulgarelli 2023). The possibility to mine alleles and use this information to create crops with enhanced roots and rhizospheres for stress tolerance is made possible by the rising accessibility of these techniques and online educational resources. Additionally, cutting-edge genome editing methods can be used to precisely manipulate target regions, and Clustered Regularly Interspaced Short Palindromic Repeats (CRISPR) technologies are available for the improvement of crop root phenotypes (Camerlengo et al. 2022; Chattopadhyay et al. 2022). Future crops will also need to harness understanding of stress memory and epigenetic responses of crops to stress to be able to help deliver plastically responsive genotypes to cope with temporally variable and extreme climatic conditions (Liu et al. 2022).

### Use of modelling in harnessing root and rhizosphere processes to mitigate and adapt to climate change

Models are needed to provide predictions similar to weather forecasts, but with variables relevant for agricultural and ecosystem functions such as plant available water, nutrient uptake, biomass and yield, soil carbon sequestration, N losses, or gaseous emissions. As climate change might require new plant/root ideotypes faster than can be provided with breeding alone, model-assisted root ideotyping for future climate scenarios (including genetic/gene level) will gain in importance. The emergent behaviour on the larger scales might not always be obvious from individual rhizosphere studies and modelling will help in upscaling of rhizosphere processes to plant and field/plot scale, and in upscaling of individual field simulations to estimate regional/national/global effects. Modelling is therefore implicit in the delivery of all the above future research priorities.

## Data Availability

As a review article there are no novel data presented in the manuscri.
